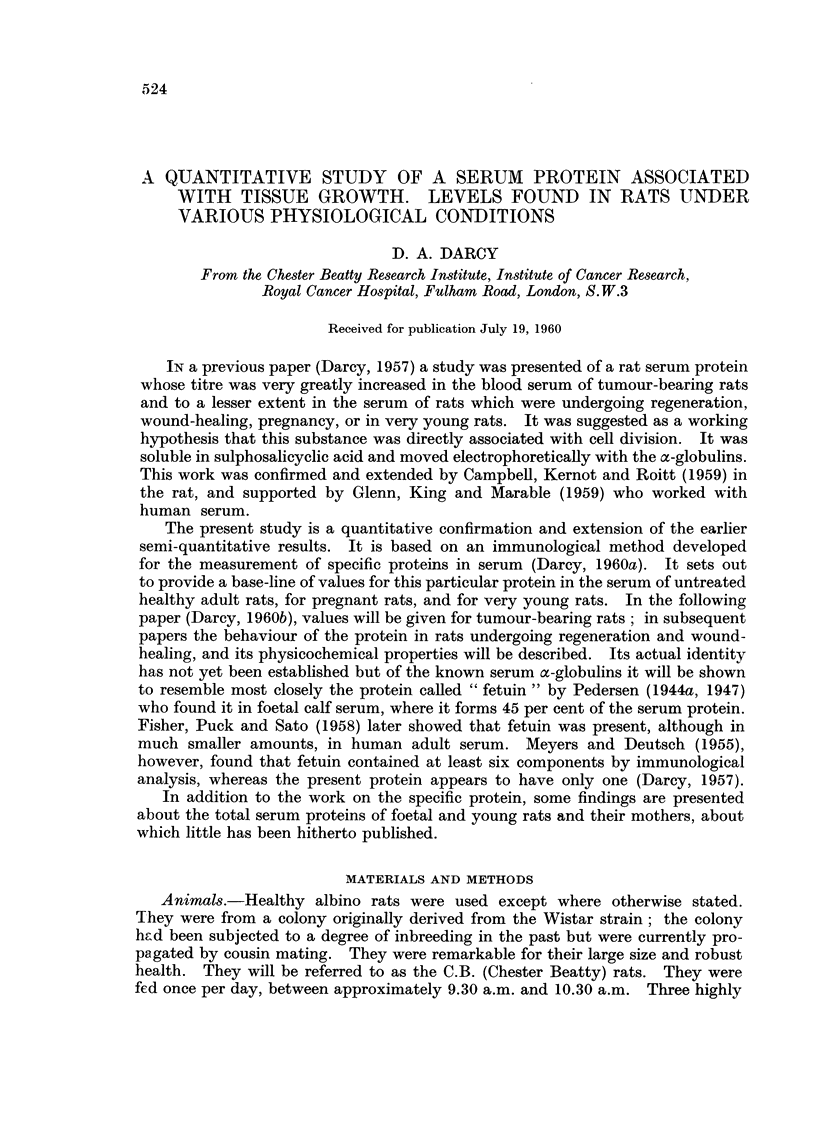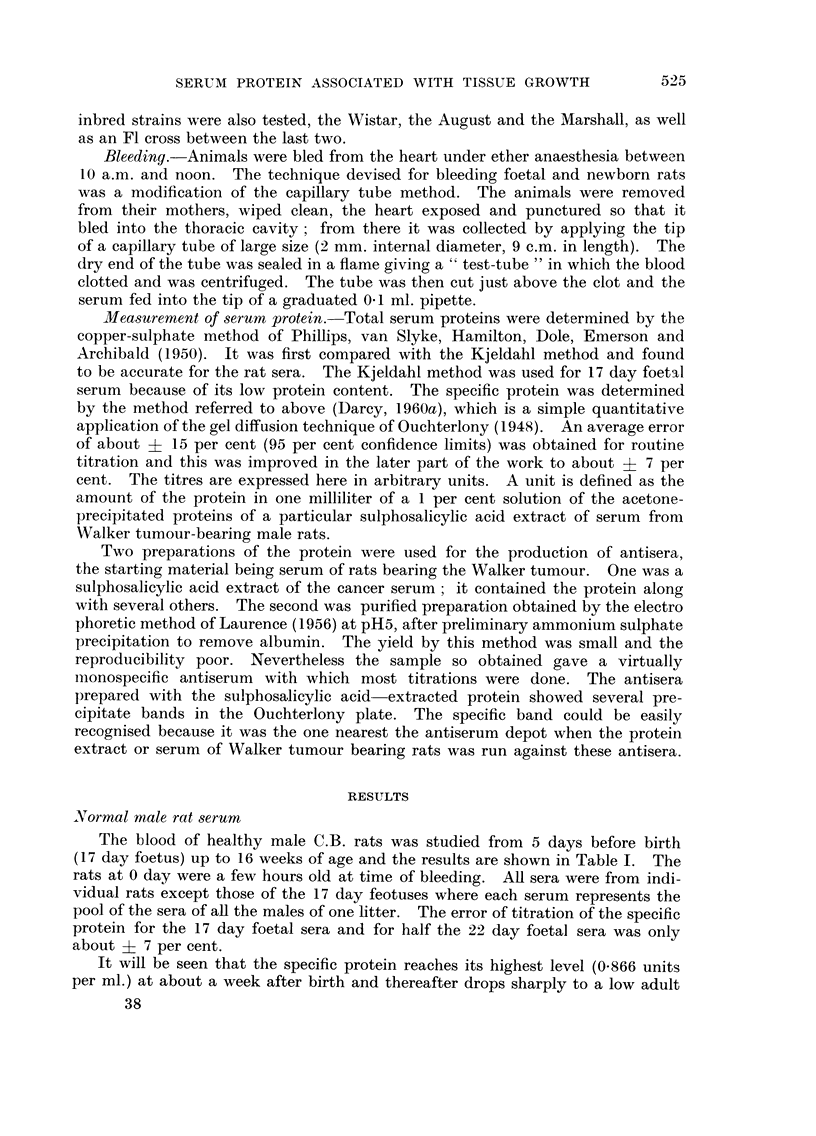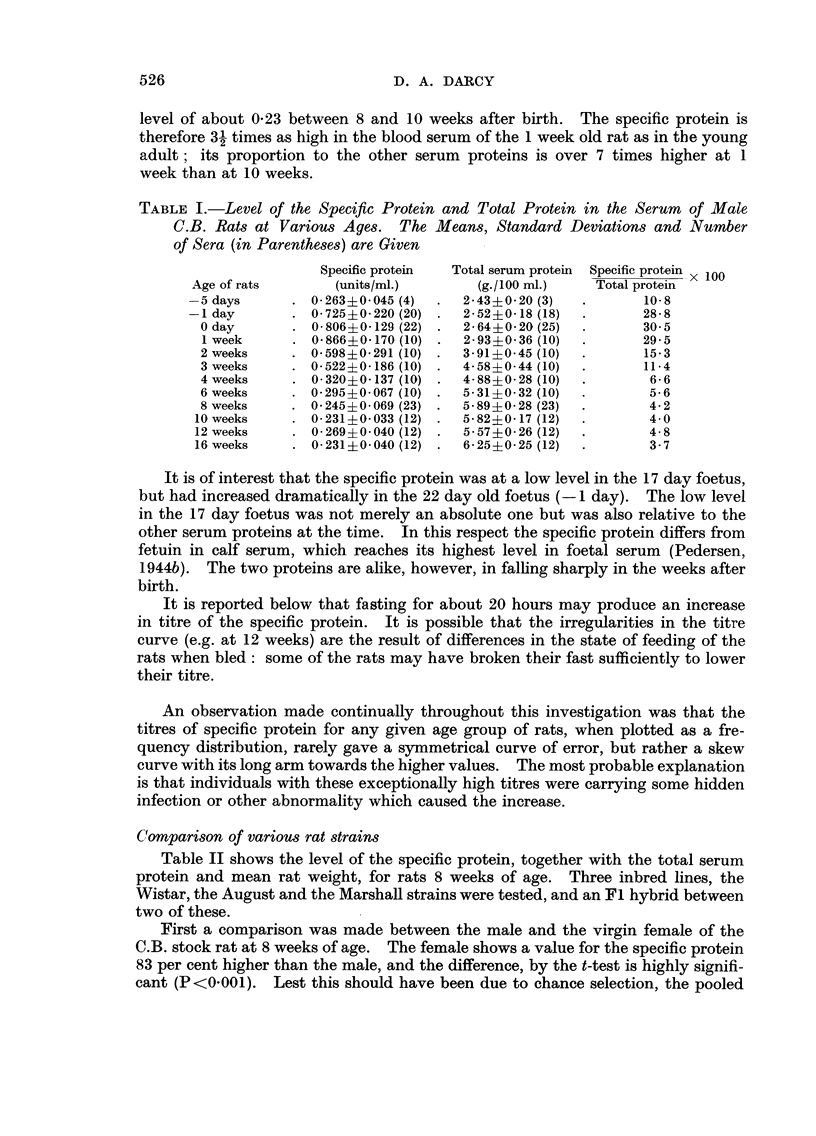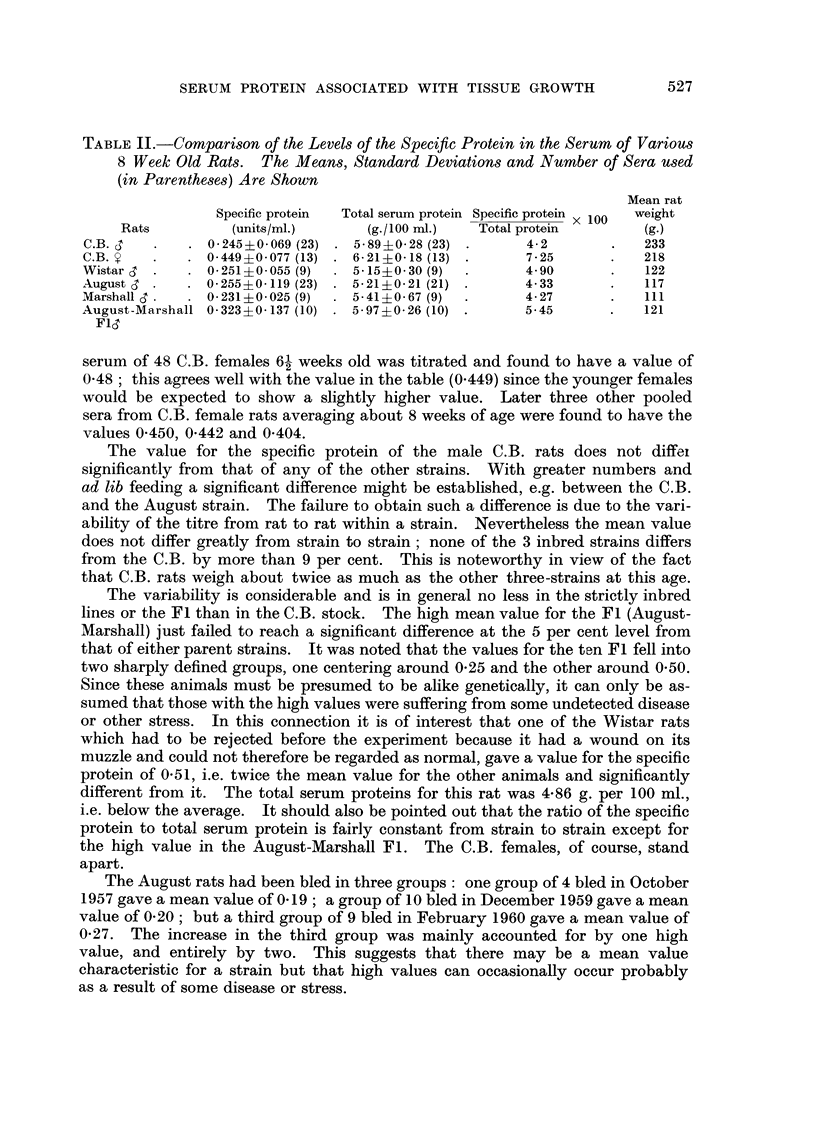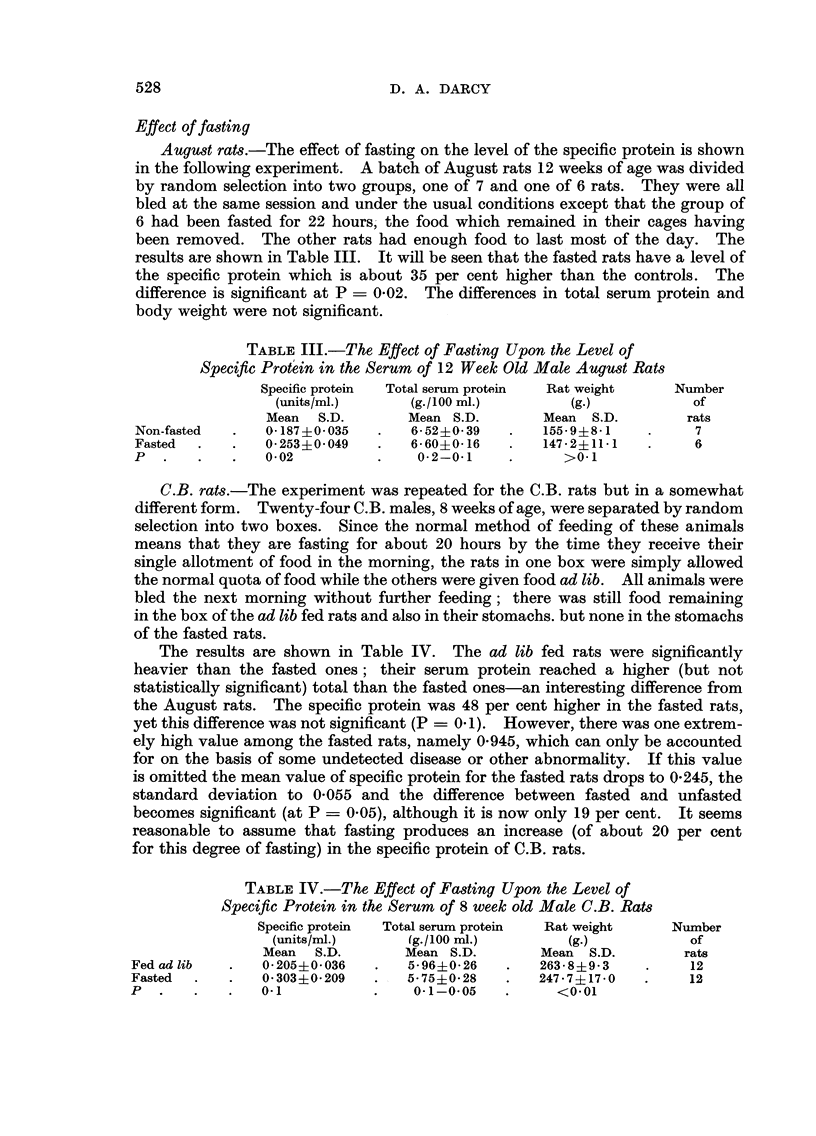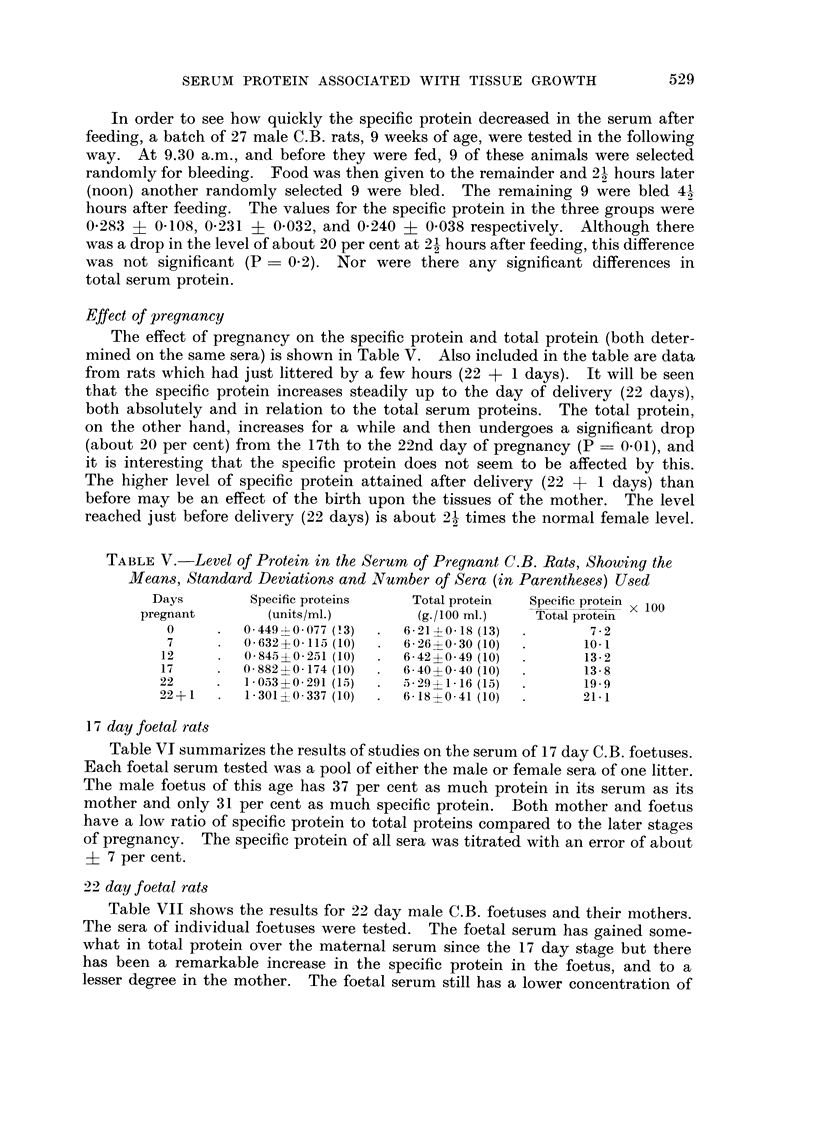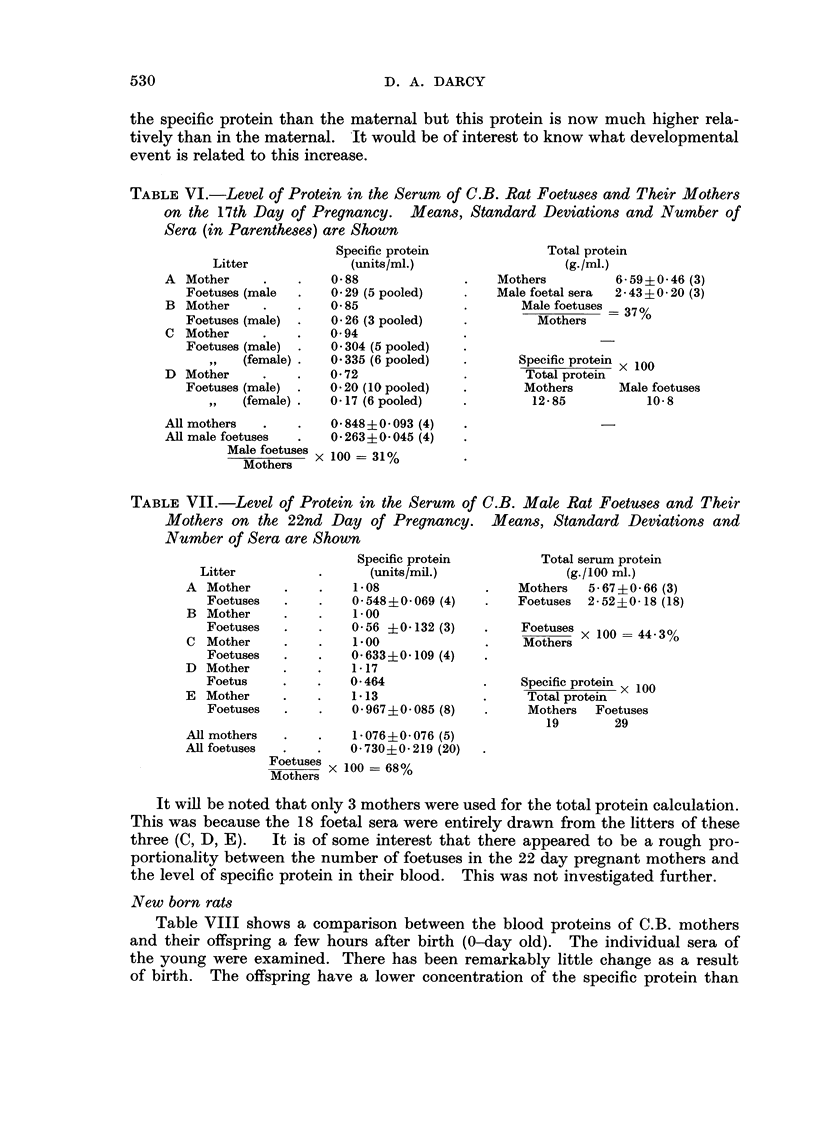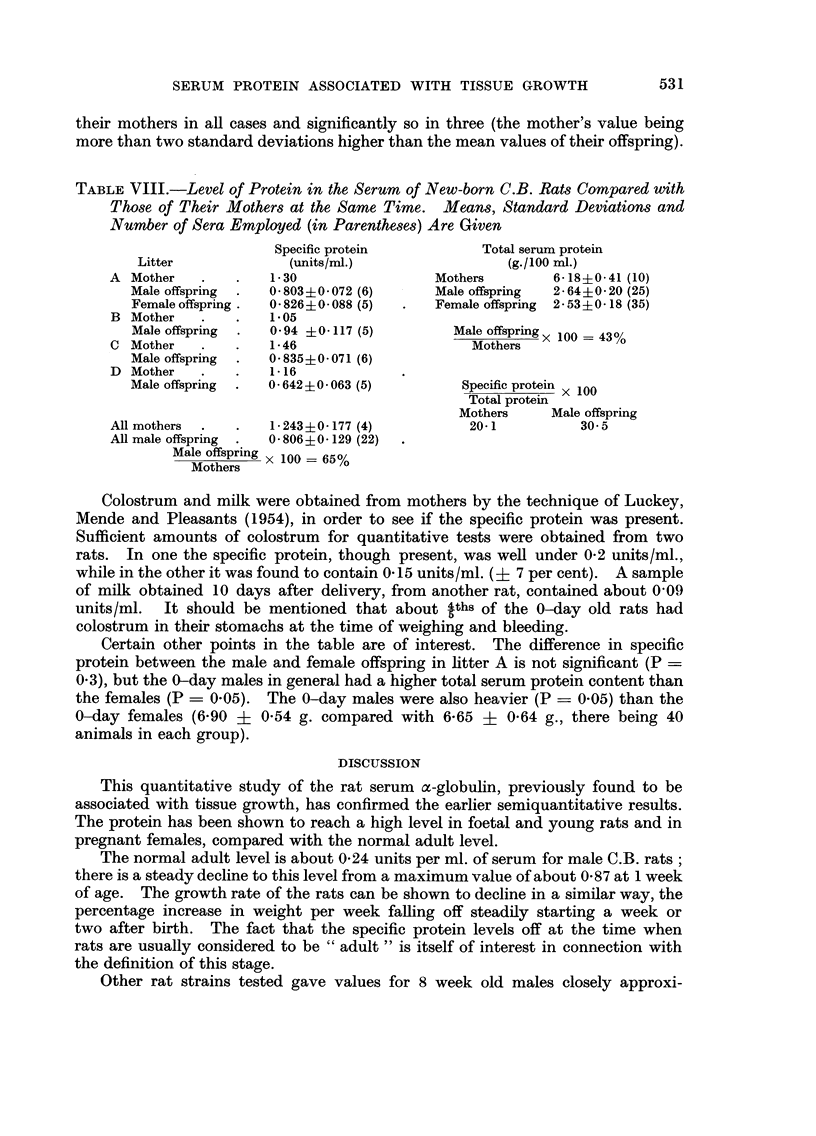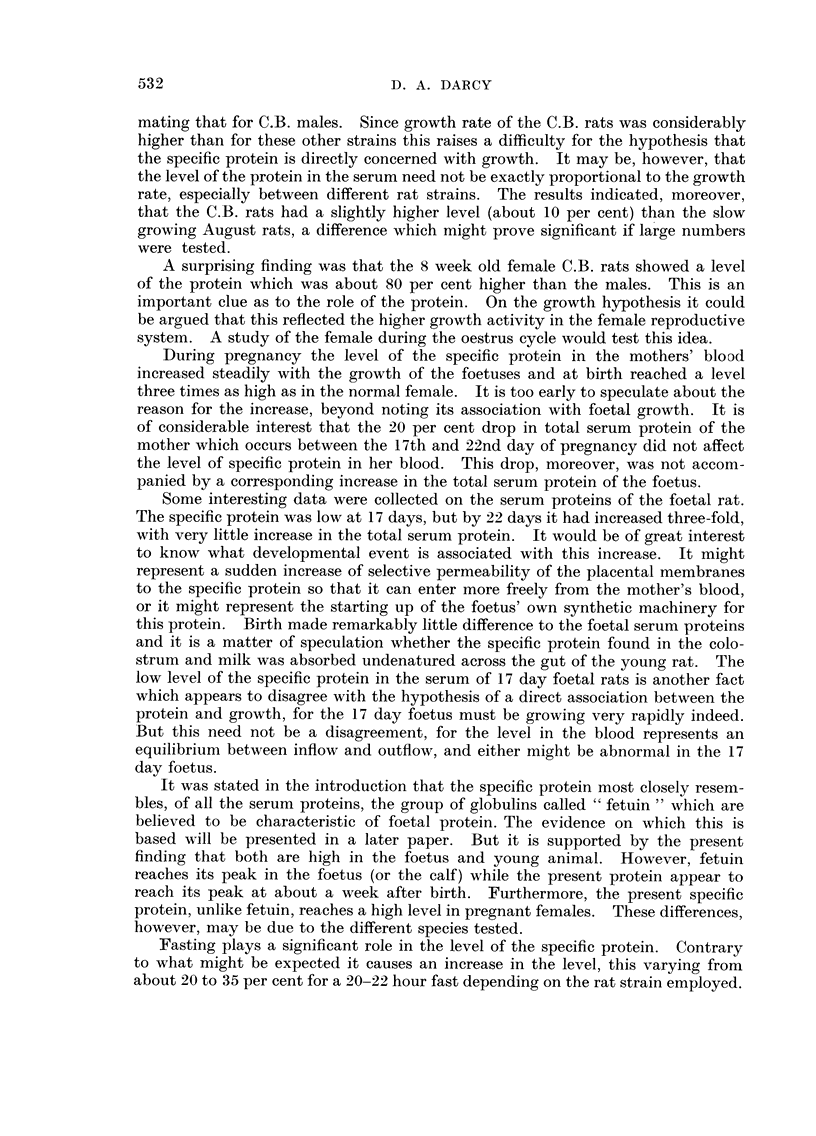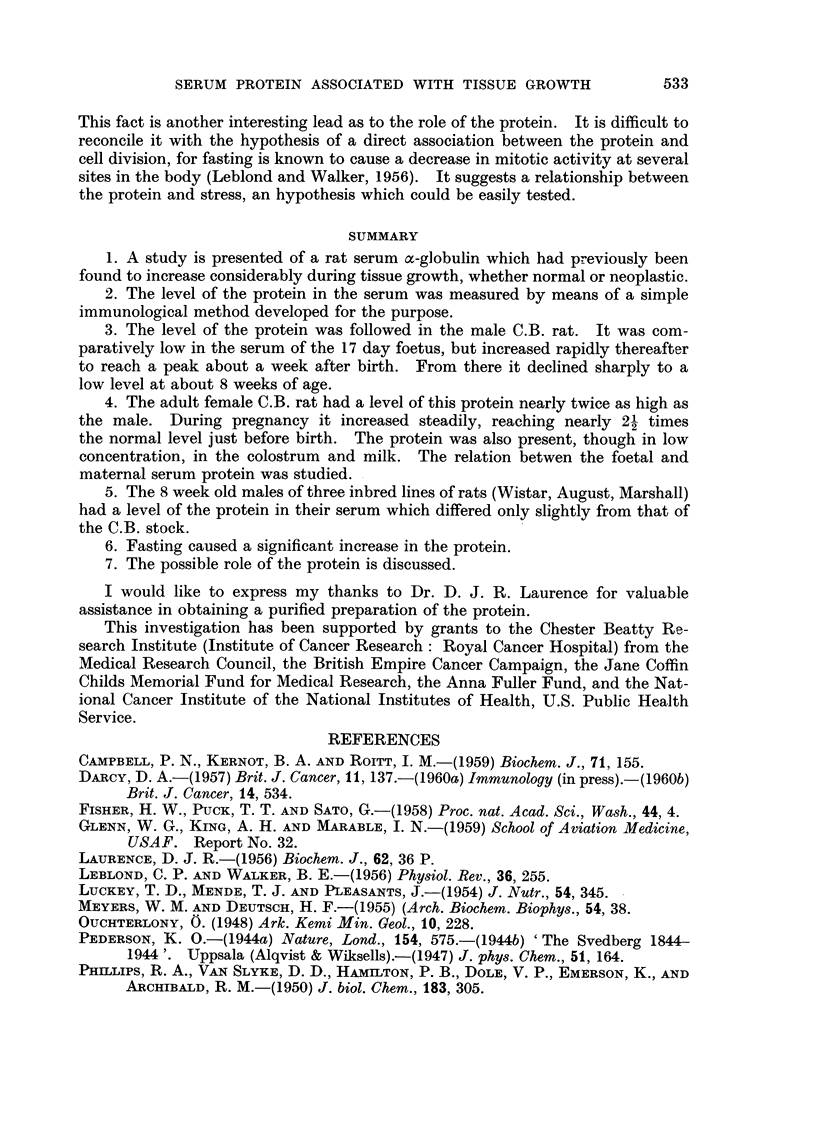# A Quantitative Study of a Serum Protein Associated with Tissue Growth. Levels found in Rats under Various Physiological Conditions

**DOI:** 10.1038/bjc.1960.59

**Published:** 1960-09

**Authors:** D. A. Darcy


### A QUANTITATIVE STUDY OF A SERUM PROTEIN ASSOCIATED WITH TISSUE GROWTH. LEVELS FOUND IN RATS UNDER VARIOUS PHYSIOLOGICAL CONDITIONS

D. A. DARCY

*From the Chester Beatty Research Institute, Institute of Cancer Research, Royal Cancer Hospital, Fulham Road, London, S.W.3*

Received for publication July 19, 1960

In a previous paper (Darcy, 1957) a study was presented of a rat serum protein whose titre was very greatly increased in the blood serum of tumour-bearing rats and to a lesser extent in the serum of rats which were undergoing regeneration, wound-healing, pregnancy, or in very young rats. It was suggested as a working hypothesis that this substance was directly associated with cell division. It was soluble in sulphosalicyclic acid and moved electrophoretically with the $\alpha$-globulins. This work was confirmed and extended by Campbell, Kernot and Roitt (1959) in the rat, and supported by Glenn, King and Marable (1959) who worked with human serum.

The present study is a quantitative confirmation and extension of the earlier semi-quantitative results. It is based on an immunological method developed for the measurement of specific proteins in serum (Darcy, 1960*a*). It sets out to provide a base-line of values for this particular protein in the serum of untreated healthy adult rats, for pregnant rats, and for very young rats. In the following paper (Darcy, 1960*b*), values will be given for tumour-bearing rats ; in subsequent papers the behaviour of the protein in rats undergoing regeneration and wound-healing, and its physicochemical properties will be described. Its actual identity has not yet been established but of the known serum $\alpha$-globulins it will be shown to resemble most closely the protein called " fetuin " by Pedersen (1944*a*, 1947) who found it in foetal calf serum, where it forms 45 per cent of the serum protein. Fisher, Puck and Sato (1958) later showed that fetuin was present, although in much smaller amounts, in human adult serum. Meyers and Deutsch (1955), however, found that fetuin contained at least six components by immunological analysis, whereas the present protein appears to have only one (Darcy, 1957).

In addition to the work on the specific protein, some findings are presented about the total serum proteins of foetal and young rats and their mothers, about which little has been hitherto published.

### MATERIALS AND METHODS

*Animals.*—Healthy albino rats were used except where otherwise stated. They were from a colony originally derived from the Wistar strain ; the colony had been subjected to a degree of inbreeding in the past but were currently propagated by cousin mating. They were remarkable for their large size and robust health. They will be referred to as the C.B. (Chester Beatty) rats. They were fed once per day, between approximately 9.30 a.m. and 10.30 a.m. Three highly inbred strains were also tested, the Wistar, the August and the Marshall, as well as an Fl cross between the last two.

*Bleeding.*—Animals were bled from the heart under ether anaesthesia between 10 a.m. and noon. The technique devised for bleeding foetal and newborn rats was a modification of the capillary tube method. The animals were removed from their mothers, wiped clean, the heart exposed and punctured so that it bled into the thoracic cavity ; from there it was collected by applying the tip of a capillary tube of large size (2 mm. internal diameter, 9 c.m. in length). The dry end of the tube was sealed in a flame giving a " test-tube " in which the blood clotted and was centrifuged. The tube was then cut just above the clot and the serum fed into the tip of a graduated 0·1 ml. pipette.

*Measurement of serum protein.*—Total serum proteins were determined by the copper-sulphate method of Phillips, van Slyke, Hamilton, Dole, Emerson and Archibald (1950). It was first compared with the Kjeldahl method and found to be accurate for the rat sera. The Kjeldahl method was used for 17 day foetal serum because of its low protein content. The specific protein was determined by the method referred to above (Darcy, 1960*a*), which is a simple quantitative application of the gel diffusion technique of Ouchterlony (1948). An average error of about $\pm$ 15 per cent (95 per cent confidence limits) was obtained for routine titration and this was improved in the later part of the work to about $\pm$ 7 per cent. The titres are expressed here in arbitrary units. A unit is defined as the amount of the protein in one milliliter of a 1 per cent solution of the acetone-precipitated proteins of a particular sulphosalicylic acid extract of serum from Walker tumour-bearing male rats.

Two preparations of the protein were used for the production of antisera, the starting material being serum of rats bearing the Walker tumour. One was a sulphosalicylic acid extract of the cancer serum ; it contained the protein along with several others. The second was purified preparation obtained by the electro phoretic method of Laurence (1956) at pH5, after preliminary ammonium sulphate precipitation to remove albumin. The yield by this method was small and the reproducibility poor. Nevertheless the sample so obtained gave a virtually monospecific antiserum with which most titrations were done. The antisera prepared with the sulphosalicylic acid—extracted protein showed several precipitate bands in the Ouchterlony plate. The specific band could be easily recognised because it was the one nearest the antiserum depot when the protein extract or serum of Walker tumour bearing rats was run against these antisera.

### RESULTS

### *Normal male rat serum*

The blood of healthy male C.B. rats was studied from 5 days before birth (17 day foetus) up to 16 weeks of age and the results are shown in Table I. The rats at 0 day were a few hours old at time of bleeding. All sera were from individual rats except those of the 17 day featuses where each serum represents the pool of the sera of all the males of one litter. The error of titration of the specific protein for the 17 day foetal sera and for half the 22 day foetal sera was only about $\pm$ 7 per cent.

It will be seen that the specific protein reaches its highest level (0·866 units per ml.) at about a week after birth and thereafter drops sharply to a low adult level of about 0·23 between 8 and 10 weeks after birth. The specific protein is therefore $3\frac{1}{2}$ times as high in the blood serum of the 1 week old rat as in the young adult ; its proportion to the other serum proteins is over 7 times higher at 1 week than at 10 weeks.

TABLE I.—*Level of the Specific Protein and Total Protein in the Serum of Male C.B. Rats at Various Ages. The Means, Standard Deviations and Number of Sera (in Parentheses) are Given*

| Age of rats | Specific protein (units/ml.) | Total serum protein (g./100 ml.) | $\frac{\text{Specific protein}}{\text{Total protein}} \times 100$ |
|---|---|---|---|
| −5 days | 0·263±0·045 (4) | 2·43±0·20 (3) | 10·8 |
| −1 day | 0·725±0·220 (20) | 2·52±0·18 (18) | 28·8 |
| 0 day | 0·806±0·129 (22) | 2·64±0·20 (25) | 30·5 |
| 1 week | 0·866±0·170 (10) | 2·93±0·36 (10) | 29·5 |
| 2 weeks | 0·598±0·291 (10) | 3·91±0·45 (10) | 15·3 |
| 3 weeks | 0·522±0·186 (10) | 4·58±0·44 (10) | 11·4 |
| 4 weeks | 0·320±0·137 (10) | 4·88±0·28 (10) | 6·6 |
| 6 weeks | 0·295±0·067 (10) | 5·31±0·32 (10) | 5·6 |
| 8 weeks | 0·245±0·069 (23) | 5·89±0·28 (23) | 4·2 |
| 10 weeks | 0·231±0·033 (12) | 5·82±0·17 (12) | 4·0 |
| 12 weeks | 0·269±0·040 (12) | 5·57±0·26 (12) | 4·8 |
| 16 weeks | 0·231±0·040 (12) | 6·25±0·25 (12) | 3·7 |

It is of interest that the specific protein was at a low level in the 17 day foetus, but had increased dramatically in the 22 day old foetus (—1 day). The low level in the 17 day foetus was not merely an absolute one but was also relative to the other serum proteins at the time. In this respect the specific protein differs from fetuin in calf serum, which reaches its highest level in foetal serum (Pedersen, 1944*b*). The two proteins are alike, however, in falling sharply in the weeks after birth.

It is reported below that fasting for about 20 hours may produce an increase in titre of the specific protein. It is possible that the irregularities in the titre curve (e.g. at 12 weeks) are the result of differences in the state of feeding of the rats when bled : some of the rats may have broken their fast sufficiently to lower their titre.

An observation made continually throughout this investigation was that the titres of specific protein for any given age group of rats, when plotted as a frequency distribution, rarely gave a symmetrical curve of error, but rather a skew curve with its long arm towards the higher values. The most probable explanation is that individuals with these exceptionally high titres were carrying some hidden infection or other abnormality which caused the increase.

*Comparison of various rat strains*

Table II shows the level of the specific protein, together with the total serum protein and mean rat weight, for rats 8 weeks of age. Three inbred lines, the Wistar, the August and the Marshall strains were tested, and an F1 hybrid between two of these.

First a comparison was made between the male and the virgin female of the C.B. stock rat at 8 weeks of age. The female shows a value for the specific protein 83 per cent higher than the male, and the difference, by the *t*-test is highly significant ($P < 0{\cdot}001$). Lest this should have been due to chance selection, the pooled

TABLE II.—*Comparison of the Levels of the Specific Protein in the Serum of Various 8 Week Old Rats. The Means, Standard Deviations and Number of Sera used (in Parentheses) Are Shown*

| Rats | Specific protein (units/ml.) | Total serum protein (g./100 ml.) | $\frac{\text{Specific protein}}{\text{Total protein}} \times 100$ | Mean rat weight (g.) |
|---|---|---|---|---|
| C.B. ♂ | 0·245±0·069 (23) | 5·89±0·28 (23) | 4·2 | 233 |
| C.B. ♀ | 0·449±0·077 (13) | 6·21±0·18 (13) | 7·25 | 218 |
| Wistar ♂ | 0·251±0·055 (9) | 5·15±0·30 (9) | 4·90 | 122 |
| August ♂ | 0·255±0·119 (23) | 5·21±0·21 (21) | 4·33 | 117 |
| Marshall ♂ | 0·231±0·025 (9) | 5·41±0·67 (9) | 4·27 | 111 |
| August-Marshall F1♂ | 0·323±0·137 (10) | 5·97±0·26 (10) | 5·45 | 121 |

serum of 48 C.B. females 6½ weeks old was titrated and found to have a value of 0·48 ; this agrees well with the value in the table (0·449) since the younger females would be expected to show a slightly higher value. Later three other pooled sera from C.B. female rats averaging about 8 weeks of age were found to have the values 0·450, 0·442 and 0·404.

The value for the specific protein of the male C.B. rats does not differ significantly from that of any of the other strains. With greater numbers and *ad lib* feeding a significant difference might be established, e.g. between the C.B. and the August strain. The failure to obtain such a difference is due to the variability of the titre from rat to rat within a strain. Nevertheless the mean value does not differ greatly from strain to strain ; none of the 3 inbred strains differs from the C.B. by more than 9 per cent. This is noteworthy in view of the fact that C.B. rats weigh about twice as much as the other three-strains at this age.

The variability is considerable and is in general no less in the strictly inbred lines or the F1 than in the C.B. stock. The high mean value for the F1 (August-Marshall) just failed to reach a significant difference at the 5 per cent level from that of either parent strains. It was noted that the values for the ten F1 fell into two sharply defined groups, one centering around 0·25 and the other around 0·50. Since these animals must be presumed to be alike genetically, it can only be assumed that those with the high values were suffering from some undetected disease or other stress. In this connection it is of interest that one of the Wistar rats which had to be rejected before the experiment because it had a wound on its muzzle and could not therefore be regarded as normal, gave a value for the specific protein of 0·51, i.e. twice the mean value for the other animals and significantly different from it. The total serum proteins for this rat was 4·86 g. per 100 ml., i.e. below the average. It should also be pointed out that the ratio of the specific protein to total serum protein is fairly constant from strain to strain except for the high value in the August-Marshall F1. The C.B. females, of course, stand apart.

The August rats had been bled in three groups : one group of 4 bled in October 1957 gave a mean value of 0·19 ; a group of 10 bled in December 1959 gave a mean value of 0·20 ; but a third group of 9 bled in February 1960 gave a mean value of 0·27. The increase in the third group was mainly accounted for by one high value, and entirely by two. This suggests that there may be a mean value characteristic for a strain but that high values can occasionally occur probably as a result of some disease or stress.

*Effect of fasting*

*August rats.*—The effect of fasting on the level of the specific protein is shown in the following experiment. A batch of August rats 12 weeks of age was divided by random selection into two groups, one of 7 and one of 6 rats. They were all bled at the same session and under the usual conditions except that the group of 6 had been fasted for 22 hours, the food which remained in their cages having been removed. The other rats had enough food to last most of the day. The results are shown in Table III. It will be seen that the fasted rats have a level of the specific protein which is about 35 per cent higher than the controls. The difference is significant at P = 0·02. The differences in total serum protein and body weight were not significant.

TABLE III.—*The Effect of Fasting Upon the Level of Specific Protein in the Serum of* 12 *Week Old Male August Rats*

| | Specific protein (units/ml.) Mean S.D. | Total serum protein (g./100 ml.) Mean S.D. | Rat weight (g.) Mean S.D. | Number of rats |
|---|---|---|---|---|
| Non-fasted | 0·187±0·035 | 6·52±0·39 | 155·9±8·1 | 7 |
| Fasted | 0·253±0·049 | 6·60±0·16 | 147·2±11·1 | 6 |
| *P* | 0·02 | 0·2–0·1 | >0·1 | |

*C.B. rats.*—The experiment was repeated for the C.B. rats but in a somewhat different form. Twenty-four C.B. males, 8 weeks of age, were separated by random selection into two boxes. Since the normal method of feeding of these animals means that they are fasting for about 20 hours by the time they receive their single allotment of food in the morning, the rats in one box were simply allowed the normal quota of food while the others were given food *ad lib*. All animals were bled the next morning without further feeding ; there was still food remaining in the box of the *ad lib* fed rats and also in their stomachs. but none in the stomachs of the fasted rats.

The results are shown in Table IV. The *ad lib* fed rats were significantly heavier than the fasted ones ; their serum protein reached a higher (but not statistically significant) total than the fasted ones—an interesting difference from the August rats. The specific protein was 48 per cent higher in the fasted rats, yet this difference was not significant (P = 0·1). However, there was one extremely high value among the fasted rats, namely 0·945, which can only be accounted for on the basis of some undetected disease or other abnormality. If this value is omitted the mean value of specific protein for the fasted rats drops to 0·245, the standard deviation to 0·055 and the difference between fasted and unfasted becomes significant (at P = 0·05), although it is now only 19 per cent. It seems reasonable to assume that fasting produces an increase (of about 20 per cent for this degree of fasting) in the specific protein of C.B. rats.

TABLE IV.—*The Effect of Fasting Upon the Level of Specific Protein in the Serum of* 8 *week old Male C.B. Rats*

| | Specific protein (units/ml.) Mean S.D. | Total serum protein (g./100 ml.) Mean S.D. | Rat weight (g.) Mean S.D. | Number of rats |
|---|---|---|---|---|
| Fed *ad lib* | 0·205±0·036 | 5·96±0·26 | 263·8±9·3 | 12 |
| Fasted | 0·303±0·209 | 5·75±0·28 | 247·7±17·0 | 12 |
| *P* | 0·1 | 0·1–0·05 | <0·01 | |

In order to see how quickly the specific protein decreased in the serum after feeding, a batch of 27 male C.B. rats, 9 weeks of age, were tested in the following way. At 9.30 a.m., and before they were fed, 9 of these animals were selected randomly for bleeding. Food was then given to the remainder and 2½ hours later (noon) another randomly selected 9 were bled. The remaining 9 were bled 4½ hours after feeding. The values for the specific protein in the three groups were 0·283 ± 0·108, 0·231 ± 0·032, and 0·240 ± 0·038 respectively. Although there was a drop in the level of about 20 per cent at 2½ hours after feeding, this difference was not significant ($P = 0{\cdot}2$). Nor were there any significant differences in total serum protein.

*Effect of pregnancy*

The effect of pregnancy on the specific protein and total protein (both determined on the same sera) is shown in Table V. Also included in the table are data from rats which had just littered by a few hours (22 + 1 days). It will be seen that the specific protein increases steadily up to the day of delivery (22 days), both absolutely and in relation to the total serum proteins. The total protein, on the other hand, increases for a while and then undergoes a significant drop (about 20 per cent) from the 17th to the 22nd day of pregnancy ($P = 0{\cdot}01$), and it is interesting that the specific protein does not seem to be affected by this. The higher level of specific protein attained after delivery (22 + 1 days) than before may be an effect of the birth upon the tissues of the mother. The level reached just before delivery (22 days) is about 2½ times the normal female level.

TABLE V.—*Level of Protein in the Serum of Pregnant C.B. Rats, Showing the Means, Standard Deviations and Number of Sera (in Parentheses) Used*

| Days pregnant | Specific proteins (units/ml.) | Total protein (g./100 ml.) | $\frac{\text{Specific protein}}{\text{Total protein}} \times 100$ |
|---|---|---|---|
| 0 | 0·449±0·077 (13) | 6·21±0·18 (13) | 7·2 |
| 7 | 0·632±0·115 (10) | 6·26±0·30 (10) | 10·1 |
| 12 | 0·845±0·251 (10) | 6·42±0·49 (10) | 13·2 |
| 17 | 0·882±0·174 (10) | 6·40±0·40 (10) | 13·8 |
| 22 | 1·053±0·291 (15) | 5·29±1·16 (15) | 19·9 |
| 22+1 | 1·301±0·337 (10) | 6·18±0·41 (10) | 21·1 |

17 *day foetal rats*

Table VI summarizes the results of studies on the serum of 17 day C.B. foetuses. Each foetal serum tested was a pool of either the male or female sera of one litter. The male foetus of this age has 37 per cent as much protein in its serum as its mother and only 31 per cent as much specific protein. Both mother and foetus have a low ratio of specific protein to total proteins compared to the later stages of pregnancy. The specific protein of all sera was titrated with an error of about ± 7 per cent.

22 *day foetal rats*

Table VII shows the results for 22 day male C.B. foetuses and their mothers. The sera of individual foetuses were tested. The foetal serum has gained somewhat in total protein over the maternal serum since the 17 day stage but there has been a remarkable increase in the specific protein in the foetus, and to a lesser degree in the mother. The foetal serum still has a lower concentration of the specific protein than the maternal but this protein is now much higher relatively than in the maternal. It would be of interest to know what developmental event is related to this increase.

TABLE VI.—*Level of Protein in the Serum of C.B. Rat Foetuses and Their Mothers on the 17th Day of Pregnancy. Means, Standard Deviations and Number of Sera (in Parentheses) are Shown*

| Litter | | Specific protein (units/ml.) | Total protein (g./ml.) | |
|---|---|---|---|---|
| A | Mother | 0·88 | Mothers | 6·59±0·46 (3) |
| | Foetuses (male | 0·29 (5 pooled) | Male foetal sera | 2·43±0·20 (3) |
| B | Mother | 0·85 | $\frac{\text{Male foetuses}}{\text{Mothers}} = 37\%$ | |
| | Foetuses (male) | 0·26 (3 pooled) | | |
| C | Mother | 0·94 | | |
| | Foetuses (male) | 0·304 (5 pooled) | — | |
| | ,, (female) | 0·335 (6 pooled) | | |
| D | Mother | 0·72 | $\frac{\text{Specific protein}}{\text{Total protein}} \times 100$ | |
| | Foetuses (male) | 0·20 (10 pooled) | Mothers | Male foetuses |
| | ,, (female) | 0·17 (6 pooled) | 12·85 | 10·8 |
| All mothers | | 0·848±0·093 (4) | — | |
| All male foetuses | | 0·263±0·045 (4) | | |
| $\frac{\text{Male foetuses}}{\text{Mothers}} \times 100 = 31\%$ | | | | |

TABLE VII.—*Level of Protein in the Serum of C.B. Male Rat Foetuses and Their Mothers on the 22nd Day of Pregnancy. Means, Standard Deviations and Number of Sera are Shown*

| Litter | | Specific protein (units/mil.) | Total serum protein (g./100 ml.) | |
|---|---|---|---|---|
| A | Mother | 1·08 | Mothers | 5·67±0·66 (3) |
| | Foetuses | 0·548±0·069 (4) | Foetuses | 2·52±0·18 (18) |
| B | Mother | 1·00 | | |
| | Foetuses | 0·56 ±0·132 (3) | $\frac{\text{Foetuses}}{\text{Mothers}} \times 100 = 44{\cdot}3\%$ | |
| C | Mother | 1·00 | | |
| | Foetuses | 0·633±0·109 (4) | | |
| D | Mother | 1·17 | | |
| | Foetus | 0·464 | $\frac{\text{Specific protein}}{\text{Total protein}} \times 100$ | |
| E | Mother | 1·13 | | |
| | Foetuses | 0·967±0·085 (8) | Mothers | Foetuses |
| | | | 19 | 29 |
| All mothers | | 1·076±0·076 (5) | | |
| All foetuses | | 0·730±0·219 (20) | | |
| $\frac{\text{Foetuses}}{\text{Mothers}} \times 100 = 68\%$ | | | | |

It will be noted that only 3 mothers were used for the total protein calculation. This was because the 18 foetal sera were entirely drawn from the litters of these three (C, D, E). It is of some interest that there appeared to be a rough proportionality between the number of foetuses in the 22 day pregnant mothers and the level of specific protein in their blood. This was not investigated further.

*New born rats*

Table VIII shows a comparison between the blood proteins of C.B. mothers and their offspring a few hours after birth (0–day old). The individual sera of the young were examined. There has been remarkably little change as a result of birth. The offspring have a lower concentration of the specific protein than their mothers in all cases and significantly so in three (the mother's value being more than two standard deviations higher than the mean values of their offspring).

TABLE VIII.—*Level of Protein in the Serum of New-born C.B. Rats Compared with Those of Their Mothers at the Same Time. Means, Standard Deviations and Number of Sera Employed (in Parentheses) Are Given*

| Litter | | Specific protein (units/ml.) |
|---|---|---|
| A | Mother | 1·30 |
| | Male offspring | 0·803±0·072 (6) |
| | Female offspring | 0·826±0·088 (5) |
| B | Mother | 1·05 |
| | Male offspring | 0·94 ±0·117 (5) |
| C | Mother | 1·46 |
| | Male offspring | 0·835±0·071 (6) |
| D | Mother | 1·16 |
| | Male offspring | 0·642±0·063 (5) |
| All mothers | | 1·243±0·177 (4) |
| All male offspring | | 0·806±0·129 (22) |

$$\frac{\text{Male offspring}}{\text{Mothers}} \times 100 = 65\%$$

| | Total serum protein (g./100 ml.) |
|---|---|
| Mothers | 6·18±0·41 (10) |
| Male offspring | 2·64±0·20 (25) |
| Female offspring | 2·53±0·18 (35) |

$$\frac{\text{Male offspring}}{\text{Mothers}} \times 100 = 43\%$$

$$\frac{\text{Specific protein}}{\text{Total protein}} \times 100$$

| Mothers | Male offspring |
|---|---|
| 20·1 | 30·5 |

Colostrum and milk were obtained from mothers by the technique of Luckey, Mende and Pleasants (1954), in order to see if the specific protein was present. Sufficient amounts of colostrum for quantitative tests were obtained from two rats. In one the specific protein, though present, was well under 0·2 units/ml., while in the other it was found to contain 0·15 units/ml. (± 7 per cent). A sample of milk obtained 10 days after delivery, from another rat, contained about 0·09 units/ml. It should be mentioned that about $\frac{4}{5}$ths of the 0–day old rats had colostrum in their stomachs at the time of weighing and bleeding.

Certain other points in the table are of interest. The difference in specific protein between the male and female offspring in litter A is not significant (P = 0·3), but the 0–day males in general had a higher total serum protein content than the females (P = 0·05). The 0–day males were also heavier (P = 0·05) than the 0–day females (6·90 ± 0·54 g. compared with 6·65 ± 0·64 g., there being 40 animals in each group).

### DISCUSSION

This quantitative study of the rat serum α-globulin, previously found to be associated with tissue growth, has confirmed the earlier semiquantitative results. The protein has been shown to reach a high level in foetal and young rats and in pregnant females, compared with the normal adult level.

The normal adult level is about 0·24 units per ml. of serum for male C.B. rats ; there is a steady decline to this level from a maximum value of about 0·87 at 1 week of age. The growth rate of the rats can be shown to decline in a similar way, the percentage increase in weight per week falling off steadily starting a week or two after birth. The fact that the specific protein levels off at the time when rats are usually considered to be " adult " is itself of interest in connection with the definition of this stage.

Other rat strains tested gave values for 8 week old males closely approximating that for C.B. males. Since growth rate of the C.B. rats was considerably higher than for these other strains this raises a difficulty for the hypothesis that the specific protein is directly concerned with growth. It may be, however, that the level of the protein in the serum need not be exactly proportional to the growth rate, especially between different rat strains. The results indicated, moreover, that the C.B. rats had a slightly higher level (about 10 per cent) than the slow growing August rats, a difference which might prove significant if large numbers were tested.

A surprising finding was that the 8 week old female C.B. rats showed a level of the protein which was about 80 per cent higher than the males. This is an important clue as to the role of the protein. On the growth hypothesis it could be argued that this reflected the higher growth activity in the female reproductive system. A study of the female during the oestrus cycle would test this idea.

During pregnancy the level of the specific protein in the mothers' blood increased steadily with the growth of the foetuses and at birth reached a level three times as high as in the normal female. It is too early to speculate about the reason for the increase, beyond noting its association with foetal growth. It is of considerable interest that the 20 per cent drop in total serum protein of the mother which occurs between the 17th and 22nd day of pregnancy did not affect the level of specific protein in her blood. This drop, moreover, was not accompanied by a corresponding increase in the total serum protein of the foetus.

Some interesting data were collected on the serum proteins of the foetal rat. The specific protein was low at 17 days, but by 22 days it had increased three-fold, with very little increase in the total serum protein. It would be of great interest to know what developmental event is associated with this increase. It might represent a sudden increase of selective permeability of the placental membranes to the specific protein so that it can enter more freely from the mother's blood, or it might represent the starting up of the foetus' own synthetic machinery for this protein. Birth made remarkably little difference to the foetal serum proteins and it is a matter of speculation whether the specific protein found in the colostrum and milk was absorbed undenatured across the gut of the young rat. The low level of the specific protein in the serum of 17 day foetal rats is another fact which appears to disagree with the hypothesis of a direct association between the protein and growth, for the 17 day foetus must be growing very rapidly indeed. But this need not be a disagreement, for the level in the blood represents an equilibrium between inflow and outflow, and either might be abnormal in the 17 day foetus.

It was stated in the introduction that the specific protein most closely resembles, of all the serum proteins, the group of globulins called " fetuin " which are believed to be characteristic of foetal protein. The evidence on which this is based will be presented in a later paper. But it is supported by the present finding that both are high in the foetus and young animal. However, fetuin reaches its peak in the foetus (or the calf) while the present protein appear to reach its peak at about a week after birth. Furthermore, the present specific protein, unlike fetuin, reaches a high level in pregnant females. These differences, however, may be due to the different species tested.

Fasting plays a significant role in the level of the specific protein. Contrary to what might be expected it causes an increase in the level, this varying from about 20 to 35 per cent for a 20–22 hour fast depending on the rat strain employed.

This fact is another interesting lead as to the role of the protein. It is difficult to reconcile it with the hypothesis of a direct association between the protein and cell division, for fasting is known to cause a decrease in mitotic activity at several sites in the body (Leblond and Walker, 1956). It suggests a relationship between the protein and stress, an hypothesis which could be easily tested.

SUMMARY

1. A study is presented of a rat serum $\alpha$-globulin which had previously been found to increase considerably during tissue growth, whether normal or neoplastic.

2. The level of the protein in the serum was measured by means of a simple immunological method developed for the purpose.

3. The level of the protein was followed in the male C.B. rat. It was comparatively low in the serum of the 17 day foetus, but increased rapidly thereafter to reach a peak about a week after birth. From there it declined sharply to a low level at about 8 weeks of age.

4. The adult female C.B. rat had a level of this protein nearly twice as high as the male. During pregnancy it increased steadily, reaching nearly $2\frac{1}{2}$ times the normal level just before birth. The protein was also present, though in low concentration, in the colostrum and milk. The relation betwen the foetal and maternal serum protein was studied.

5. The 8 week old males of three inbred lines of rats (Wistar, August, Marshall) had a level of the protein in their serum which differed only slightly from that of the C.B. stock.

6. Fasting caused a significant increase in the protein.

7. The possible role of the protein is discussed.

I would like to express my thanks to Dr. D. J. R. Laurence for valuable assistance in obtaining a purified preparation of the protein.

This investigation has been supported by grants to the Chester Beatty Research Institute (Institute of Cancer Research : Royal Cancer Hospital) from the Medical Research Council, the British Empire Cancer Campaign, the Jane Coffin Childs Memorial Fund for Medical Research, the Anna Fuller Fund, and the National Cancer Institute of the National Institutes of Health, U.S. Public Health Service.

### REFERENCES

CAMPBELL, P. N., KERNOT, B. A. AND ROITT, I. M.—(1959) *Biochem. J.*, **71**, 155.

DARCY, D. A.—(1957) *Brit. J. Cancer*, **11**, 137.—(1960*a*) *Immunology* (in press).—(1960*b*) *Brit. J. Cancer*, **14**, 534.

FISHER, H. W., PUCK, T. T. AND SATO, G.—(1958) *Proc. nat. Acad. Sci., Wash.*, **44**, 4.

GLENN, W. G., KING, A. H. AND MARABLE, I. N.—(1959) *School of Aviation Medicine, USAF.* Report No. 32.

LAURENCE, D. J. R.—(1956) *Biochem. J.*, **62**, 36 P.

LEBLOND, C. P. AND WALKER, B. E.—(1956) *Physiol. Rev.*, **36**, 255.

LUCKEY, T. D., MENDE, T. J. AND PLEASANTS, J.—(1954) *J. Nutr.*, **54**, 345.

MEYERS, W. M. AND DEUTSCH, H. F.—(1955) (*Arch. Biochem. Biophys.*, **54**, 38.

OUCHTERLONY, Ö. (1948) *Ark. Kemi Min. Geol.*, **10**, 228.

PEDERSON, K. O.—(1944*a*) *Nature, Lond.*, **154**, 575.—(1944*b*) 'The Svedberg 1844–1944'. Uppsala (Alqvist & Wiksells).—(1947) *J. phys. Chem.*, **51**, 164.

PHILLIPS, R. A., VAN SLYKE, D. D., HAMILTON, P. B., DOLE, V. P., EMERSON, K., AND ARCHIBALD, R. M.—(1950) *J. biol. Chem.*, **183**, 305.